# Enhancement of Migration and Tenogenic Differentiation of Macaca Mulatta Tendon-Derived Stem Cells by Decellularized Tendon Hydrogel

**DOI:** 10.3389/fcell.2021.651583

**Published:** 2021-04-27

**Authors:** Liang-Ju Ning, Ya-Jing Zhang, Yan-Jing Zhang, Min Zhu, Wei Ding, Yan-Lin Jiang, Yi Zhang, Jing-Cong Luo, Ting-Wu Qin

**Affiliations:** ^1^Laboratory of Stem Cell and Tissue Engineering, Orthopedic Research Institute, State Key Laboratory of Biotherapy and Cancer Center, West China Hospital, Sichuan University and Collaborative Innovation Center of Biotherapy, Chengdu, China; ^2^Core Facility of West China Hospital, Sichuan University, Chengdu, China

**Keywords:** decellularized tendon, hydrogel, ECM microenvironment, Macaca mulatta, tendon-derived stem cells, migration, differentiation

## Abstract

Decellularized tendon hydrogel from human or porcine tendon has been manufactured and found to be capable of augmenting tendon repair *in vivo*. However, no studies have clarified the effect of decellularized tendon hydrogel upon stem cell behavior. In the present study, we developed a new decellularized tendon hydrogel (T-gel) from Macaca mulatta, and investigated the effect of T-gel on the proliferation, migration and tenogenic differentiation of Macaca mulatta tendon-derived stem cells (mTDSCs). The mTDSCs were first identified to have universal stem cell characteristics, including clonogenicity, expression of mesenchymal stem cell and embryonic stem cell markers, and multilineage differentiation potential. Decellularization of Macaca mulatta Achilles tendons was confirmed to be effective by histological staining and DNA quantification. The resultant T-gel exhibited highly porous structure or similar nanofibrous structure and approximately swelling ratio compared to the collagen gel (C-gel). Interestingly, stromal cell-derived factor-1 (SDF-1) and fibromodulin (Fmod) inherent in the native tendon extracellular matrix (ECM) microenvironment were retained and the values of SDF-1 and Fmod in the T-gel were significantly higher than those found in the C-gel. Compared with the C-gel, the T-gel was found to be cytocompatible with NIH-3T3 fibroblasts and displayed good histocompatibility when implanted into rat subcutaneous tissue. More importantly, it was demonstrated that the T-gel supported the proliferation of mTDSCs and significantly promoted the migration and tenogenic differentiation of mTDSCs compared to the C-gel. These findings indicated that the T-gel, with its retained nanofibrous structure and some bioactive factors of native tendon ECM microenvironment, represents a promising hydrogel for tendon regeneration.

## Introduction

Tendinopathy, also called tendinosis, is often caused by overuse or sudden stress on a tendon. Evidence from clinical and animal studies showed histopathological changes associated with tendinopathy, including degeneration and disorganization of collagen fibers, increased cellularity, as well as minimal inflammation (Khan et al., 1999; Soslowsky et al., 2000). Subsequent studies demonstrated that tendinopathy weakened the mechanical and material properties of the tendon (Arya and Kulig, 2010), and the progression of tendon fatigue damage accumulation leaded to collagen fiber rupture and eventual tendon full-thickness tearing with higher loading (Neviaser et al., 2012), which illustrated the progression from tendinopathy to full-thickness tearing. The current treatment options for tendinopathy focused on exercise-based physical therapy, other physical therapy modalities (such as ultrasound and low-level laser therapy), growth factors and stem cell treatment, and so on (Andres and Murrell, 2008). Among them, growth factors (de Vos et al., 2010; Kaux and Crielaard, 2013; Carr et al., 2015) and stem cell (Chong et al., 2007; Yuksel et al., 2016) treatments have attracted growing interest.

Extracellular matrix (ECM) hydrogels from decellularized tissues have been widely utilized to deliver exogenous stem cells (Viswanath et al., 2017; Yuan et al., 2017; Bai et al., 2019), growth factors (Seif-Naraghi et al., 2012; Xu et al., 2016; Liu et al., 2019) or other bioactive molecules for tissue repair and regeneration, which was demonstrated to support cell viability and function and enhance retention and delivery of growth factors. Besides, ECM hydrogels provide advantages such as injectability, regulable mechanical properties, good processibility as well as the ability to fill an irregularly shaped space (Wolf et al., 2012; Pati et al., 2015; Lin et al., 2018). Promisingly, previous work demonstrated that hydrogels formed from enzymatically degraded and solubilized ECM retained some of the components and bioactivity of the intact ECM (Wolf et al., 2012; Wu et al., 2015; Wang et al., 2016; Lin et al., 2018), and their degradation caused the release of many bioactive components, such as cryptic peptides, which are contribute to endogenous cell recruiting and tissue remodeling (Agrawal et al., 2011a,b). Recently, Ungerleider et al. (2020) reported that tissue specific muscle ECM hydrogel improved skeletal muscle regeneration *in vivo* over non-matched tissue source, which suggested ECM choice is a crucial consideration for optimal tissue regeneration.

Decellularized tendon ECM as a tissue-specific bioscaffold has been widely accepted as an ideal substrate for tendon regeneration. We previously developed a new and mild decellularization protocol, including physical methods and enzymatic solutions for processing canine Achilles tendons, and produced a 300 μm-thick sheet-like scaffold of decellularized tendon slices (DTSs), which retained more than 93% of fibromodulin (Fmod) and biglycan (Bgn) as well as four growth factors (TGF-β1, IGF-1, VEGF, and CTGF) of the native tendon (Ning et al., 2012). Also, the DTSs that maintained the native tendon ECM microenvironment cues, promoted stem cell proliferation and tenogenic differentiation (Ning et al., 2015). Three-dimensional (3D) collagen scaffold supplemented with tendon ECM was found to significantly increase proliferation and tenogenic differentiation of human adipose-derived stem cells (Yang et al., 2013). Promisingly, an injectable tendon hydrogel from decellularized human tendon were designed and found to hold the distinctive composition specific for tendon ECM, and displayed good biocompatibility (Farnebo et al., 2014). Then, this decellularized tendon hydrogel was demonstrated to be capable of augmenting tendon repair in rat Achilles tendon injury model as well as chronic rotator cuff injury model (Kim et al., 2014; Crowe et al., 2016). However, no studies have clarified the effect of decellularized tendon hydrogel upon stem cell behavior.

Due to their highly similar to humans in terms of genetics and physiology, the rhesus monkeys (Macaca mulatta) have become the most widely used non-human primate in basic and applied biomedical research (Gibbs et al., 2007). In this study, we developed a new decellularized tendon hydrogel (T-gel) from Macaca mulatta, and investigated the effect of T-gel on the proliferation, migration and tenogenic differentiation of Macaca mulatta tendon-derived stem cells (mTDSCs).

## Materials and Methods

### Macaca Mulatta TDSCs Isolation, Culture, and Identification

The Achilles tendons were harvested from adult Macaca mulatta within 2 h of euthanasia, which were gathered from the West China-Frontier Pharma Tech (Chengdu, China). The isolation of mTDSCs was performed according to our previously published protocol (Ning et al., 2015). Using the same protocol, we isolated and cultured mTDSCs from Macaca mulatta Achilles tendons. Fresh culture medium consisting of Dulbecco’s modified Eagle’s medium (DMEM, Gibco) supplemented with 20% fetal bovine serum (FBS), 100 μg/ml streptomycin, 100 U/ml penicillin and 2 mM L-glutamine (all from Invitrogen, Carlsbad, CA), was changed every other day. Cells at passage 3 (P3) were utilized in the subsequent experiments.

To determine the self-renewal potential of mTDSCs, the cells were seeded at 500 cells or 1,000 cells in 25-cm^2^ flasks to form colonies. After 10 days, the cell colonies were stained using 0.5% crystal violet (Sigma). The number of all colonies with diameters >2 mm was counted.

For flow cytometry analysis of cell surface antigens, mTDSCs (5 × 10^5^) were incubated with 1 μg of phycoerythrin (PE)-Cy^TM^7-conjugated mouse anti-human CD73, APC-conjugated mouse anti-human CD90, PE-conjugated mouse anti-human CD105 (BD), PE-conjugated mouse anti-human CD105 (BD) or FITC-conjugated mouse anti-human CD34 (Santa Cruz Biotechnology) and CD45 (BD) for 30 min at 4°C. After washing with phosphate-buffered saline (PBS) by centrifugation at 1,200 rpm for 5 min, the stained cells were resuspended in 200 μl of ice-cold PBS and detected by the Cytomics FC500 MCL Flow Cytometer (Beckman Coulter).

Immunofluorescent staining was performed to examine the following stem cell markers: Nanog, octamer-binding transcription factor 4 (Oct-4) and stage-specific embryonic antigen-1 (SSEA-1). mTDSCs (2 × 10^4^) were seeded on the 24 × 24 mm^2^ glass coverslips and cultured with growth medium for 3 days. The cells were then fixed in 4% paraformaldehyde for 15 min at room temperature and permeabilized with 0.5% Triton X-100. Fixed cells were washed with PBS and blocked for 30 min with 1% BSA, and then incubated with primary antibody at 4°C overnight. The primary antibodies and the titers used were as follows: rabbit anti-human Nanog (1:50, Abcam), rabbit anti-human Oct-4 (1:50, Abcam), or mouse anti-human SSEA-1 (1:50, Millpore). After washing the cells with PBS, Cy3-conjugated goat anti-rabbit IgG secondary antibody (1:200) was applied to Nanog and Oct-4 and Alexa Fluor^®^ 488-conjugated goat anti-mouse IgG antibody (1:50) (all from Jackson ImmunoResearch Laboratories, PA) was used for SSEA-1 at room temperature for 1 h. Finally, the nuclei were counter-stained with 4,6-diamidino-2-phenylindole (DAPI, Sigma). The images were observed and taken with fluorescence microscopy (Olympus, Japan).

The multidifferentiation potential of mTDSCs was tested *in vitro* for the osteogenic, adipogenic, and chondrogenic lineages as described previously (Ning et al., 2015). For osteogenic differentiation, mTDSCs (3 × 10^3^/cm^2^) were cultured in basic growth medium (containing 10% FBS, 100 μg/ml streptomycin and 100 U/ml penicillin in DMEM- low glucose) plus 0.1 μM dexamethasone, 10 mM b-glycerol phosphate, and 100 μg/ml ascorbic acid for 28 days. For adipogenic differentiation, mTDSCs (5 × 10^3^/cm^2^) were cultured in adipogenic induction medium consisting of basic growth medium supplemented with 0.5 μM dexamethasone, 50 μM indomethacin, 50 μM isobutylmethylxanthine (IBMX) and 10 μg/ml insulin for 14 days. For chondrogenic differentiation, a pellet culture system was used (Rui et al., 2010). mTDSCs (3 × 10^5^) were pelleted into a micromass by centrifugation at 1,200 rpm for 10 min in a 15-ml conical polypropylene tube and cultured in chondrogenic induction medium consisting of basic growth medium added with 0.1 μM dexamethasone, 10 ng/ml TGF-β3, 40 μg/ml proline, 100 μg/ml sodium pyruvate, 50 μg/ml ascorbate 2-phosphate and 1% ITS + Premix (BD) for 21 days. To determine the osteogenesis, adipogenesis and chondrogenesis of mTDSCs, Alizarin Red S, Oil Red O and Safranin O/Fast Green staining were performed, respectively.

### Preparation of the T-gel

The DTSs from Macaca mulatta Achilles tendons were prepared using our previously published protocol (Ning et al., 2012). In brief, the harvested Achilles tendons were firstly trimmed into segments roughly 2 cm in length and then subjected to repetitive freeze/thaw treatment, frozen section with a 300 μm thickness, as well as nuclease treatment for 12 h at 37°C. The tendon segments were only performed to longitudinally sliced into thicknesses of 300 μm, as native tendon slices (NTSs). Finally, all tendon slices were washed three times with PBS for 30 min each. The decellularization effectiveness were determined by hematoxylin and eosin (H&E) and DAPI staining, as well as DNA quantification assays.

The T-gel was prepared using a previously published protocol with some modifications (Farnebo et al., 2014). Lyophilized DTSs were cryomilled using a Mixer Mill (Retsch, MM400, Germany) after precooled in liquid nitrogen for 5 min. The milled DTSs powder was digested in a solution of 1 mg/ml pepsin (Sigma) in 0.02 M HCl such that the final concentration of decellularized tendon pre-gel was 20 or 30 mg/ml. The DTSs powder was digested for 24 h at 4°C under constant stirring. While cooled on ice, the pH was neutralized to 7.4 by adding 0.2 M NaOH (1/10 of original digest volume), and salt concentration was adjusted by adding 10 × PBS (1/10 of final neutralized volume). Then, the final mixture (pre-gel) was allowed to gel formation for 1 h at 37°C. Rat-tail tendon collagen type I gel (C-gel, 2 mg/ml; Shengyou, China) was prepared according to the manufacturer’s instruction and served as the control in this study.

### Characterization of the T-gel

The microstructure of the T-gel and C-gel was visualized by scanning electron microscopy (SEM) (ZEISS EVO10, Germany). After gelation, the gel samples were directly freeze-dried at -70°C for 24 h or immediately fixed in 2.5% glutaraldehyde at 4°C for 24 h. The freeze-dried gel samples were mounted on aluminum stubs and coated with gold, then observed with SEM. The glutaraldehyde-fixed gel samples were dehydrated in graded ethanol (50, 70, 90, and 100% ethanol) for 2 h at each concentration, and then were conducted to critical point drying. After coating with gold, the gel samples were examined with SEM.

The equilibrium swelling ratios of the T-gel and C-gel were determined by the classical gravimetric method. Both gels (*n* = 3 per group) were immersed in PBS (pH = 7.4) at 37°C for 24 h to ensure that all the gels reached their equilibrium swelling states. The equilibrium swelling ratios of the different gels were calculated according to our previous work (Wang et al., 2016) by equation: swelling ratio (%) = (Ww—Wi)/Ww × 100, where Ww and Wi are the weights of the hydrogels in the equilibrium swelling state and initial gelling state, respectively.

The bioactive factors retained in the T-gel and C-gel were measured by enzyme-linked immunosorbent assay (ELISA). Soluble molecules were extracted from the T-gel and C-gel (*n* = 6 for each group) using the Radio Immunoprecipitation Assay (RIPA) Lysis Buffer (Beyotime, China) with protease inhibitor cocktail and homogenized by the High-Throughput Tissue Automatic Grinding Machine (Servicebio, China). The extracted lysates were centrifuged at 10,000 rpm for 20 min at 4°C and then the supernatant was collected. ELISA measures were performed to detect IGF-1, SDF-1 and Fmod according to the manufacturer’s instructions (IGF-1 and SDF-1, Multisciences Biotech, China; Fmod, DLdevelop, China). All samples were normalized by per ml gel.

The *in vitro* cytotoxicity of the T-gel and C-gel was evaluated using the Live/Dead staining and the cell counting kit-8 (CCK-8, Dojindo, Japan) assay. For Live/Dead staining assay, a 96-well plate was coated with 50 μl pre-T-gel or pre-C-gel (*n* = 4 for each group), then incubated at 37°C in 5% CO_2_ for 1 h. After washing with PBS, the NIH-3T3 fibroblasts were seeded on gel-coated plates at a concentration of 4 × 10^3^ cells/well and cultured with basic growth medium. After 1, 3, and 5 days of culture, cell viability was confirmed using a fluorescent live/dead viability/cytotoxicity kit (Thermo Fisher Scientific, United States) according to the manufacturer’s protocol. Live and dead cells were observed with a phase contrast fluorescence inverted microscope (Nikon, Japan). Meanwhile, at each time point, cell proliferation was assessed using the CCK-8 assay according to the manufacturer’s protocol. After incubation at 37°C for 1.5 h, the absorbance was measured at 450 nm via a Multiskan FC^®^ Microplate Photometer (Thermo Fisher Scientific, United States). Cell number was correlated to optical density (OD).

Twenty male Sprague Dawley (SD) rats (7–8 weeks old, 250–310 g) were used to evaluate the histocompatibility of the T-gel and C-gel *in vivo*. All animal procedures were approved by the Sichuan University Animal Care and Use Committee. After anesthesia, 1 ml of pre-T-gel or pre-C-gel were injected directly into the subcutaneous tissue of rats along the dorsal midline. At 3, 7, 14, and 28 days of post-injection, five rats for each time point were euthanized. The implant/skin tissue constructs were harvested and immediately fixed in 10% neutral buffered formalin for histological evaluation using H&E and Masson’s Trichrome staining. The host response to the implants was quantitatively assessed by measuring the number of inflammatory cells, including macrophages, lymphocytes, neutrophils, eosinophils, and so on. High magnification images (400×) were obtained from four randomly selected fields in each sample under a light microscope (Olympus, Japan). At each time point, the number of inflammatory cells in five different tissue samples (*n* = 5) was measured and normalized to the gel area.

### Functional Evaluation of the T-gel

Cell viability and proliferation were determined by the Live/Dead staining and CCK-8 assay. Briefly, a 96-well plate was coated with the C-gel and T-gel prior to seeding mTDSCs at a density of 4 × 10^3^ cells/well. The cell viability of mTDSCs seeded on the C-gel and T-gel over a period of 7 days was determined using the Live/Dead staining. Cell proliferation was assessed quantitatively at 1, 3, 5, and 7 days using the CCK-8 assay.

A Transwell chemotactic migration model (pore size: 8 μm, Corning, United States) was used to assess the migration capacity of mTDSCs mediated by the C-gel and T-gel. After serum-starving for 24 h, 200 μl of mTDSCs suspension (5 × 10^4^ cells/ml) was placed within the upper chamber, and 1 ml DMEM containing 10% FBS was added to 200 μl of C-gel or T-gel coated lower chamber. After 24 or 48 h of incubation, the cells that migrated to the lower side of the membrane were fixed and dyed by DAPI (Sigma). The number of migrated cells was counted in five randomly selected fields (100×) in each well.

For tenogenic differentiation analysis, the expression of tendon-related markers at the mRNA level was tested in mTDSCs cultured on the T-gel and C-gel in basic growth medium for 3, 7, or 14 days. At each time point, total RNA was isolated by lysis in TRIzol (Life Technologies). First-strand cDNA was synthesized with oligo (dT) primers using a cDNA synthesis kit (Promega) according to the manufacturer’s instructions. Quantification of mRNA expression was performed specific for scleraxis (SCX), tenomodulin (TNMD), tenascin-C (TNC), collagen types I (COL I) and III (COL III) using the Step One Plus Real-Time PCR System (Life Technologies) and values normalized to GAPDH and presented as 2^–ΔΔ*Ct*^. All primers were designed using primer 5.0 and are summarized in Table 1.

**TABLE 1:** List of primer sequences utilized for real-time Polymerase Chain Reaction.

**Genes**	**5′-3′**	**Primer sequences**	**Production size (bp)**
GAPDH	Forward	TGACCTGCCGTCTGGAAA	138
	Reverse	GGGTGTCGCTGTTGAAGT	
SCX	Forward	CGAGAACACCCAGCCCAAAC	105
	Reverse	GCCACCTCCTAACTGCGAAT	
TNMD	Forward	TCAGTGATTTGGGTCCCAGC	218
	Reverse	GGGACCACCCACTGTTCATT	
TNC	Forward	TTTCCCAGACAGATAACAGC	197
	Reverse	AGCAGAAACTCCAATCCC	
COLI	Forward	GACATCCCACCAATCACCT	118
	Reverse	CGTCATCGCACAACACCTT	
COLIII	Forward	CAGGGAACAACTTGATGG	140
	Reverse	AGTGGGATGAAGCAGAGC	

### Statistical Analysis

Data were presented as mean ± standard deviation (SD). A two-tailed Student’s *t*-test or one-way analysis of variance (ANOVA) with a *post hoc* Dunnett’s T3 test wherever applicable, was used for statistical analysis. Differences were considered statistically significant at *P* < 0.05.

## Results

### Identification and Characterization of mTDSCs

**FIGURE 1:**
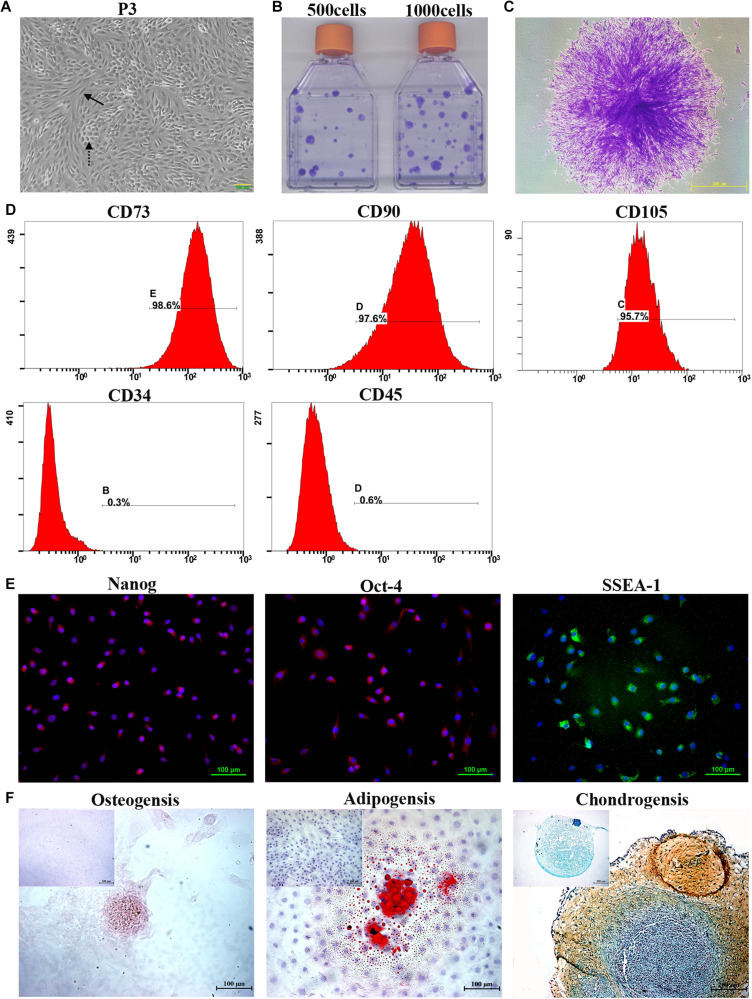
Isolation and characterization of mTDSCs. **(A)** Morphology of mTDSCs at P3. These cells were fibroblast-like morphology (solid arrow) or cobblestone-like morphology (dotted arrow). **(B**) Crystal violet staining of colonies formed by mTDSCs. **(C)** Representative image of a single clone. **(D)** Flow cytometry analysis of the expression of cell surface markers related to MSCs, hematopoietic stem cells, and endothelial cells on mTDSCs. **(E)** Immunofluorescent staining of stem cell markers for mTDSCs. Scale bar = 100 μm. **(F)** Multidifferentiation potential of mTDSCs toward osteogenesis (Scale bar = 100 μm), adipogenesis (Scale bar = 100 μm) and chondrogenesis (Scale bar = 200 μm) *in vitro*, as evidenced by Alizarin Red, Oil Red O, and Safranin O/Fast Green staining respectively. The upper left corner inset shows the mTDSCs in basic growth medium without such a multidifferentiation potential.

During the primary culture and continuous passage culture, mTDSCs mainly showed two different types of morphologies: fibroblast-like morphology and cobblestone-like morphology (Figure 1A). The colony-forming assay showed that colonies that had formed from single cells after 10 days of culture were visualized using crystal violet staining (Figures 1B,C). About 4% of mTDSCs at P3 were able to form colonies. The results of flow cytometric analysis indicated that these cells expressed a set of classic mesenchymal stem cell (MSC) markers, including CD73, CD90 and CD105 (Figure 1D). They were negative for the hematopoietic stem cell marker CD34 and for the leukocyte marker CD45, thus verifying the absence of contaminating hematopoietic cells (Figure 1D). Immunofluorescent staining of these cells showed that mTDSCs were positive for embryonic stem cells marker, such as Nanog, Oct-4 and SSEA-1 (Figure 1E). The potential of mTDSCs to undergo osteogenesis, adipogenesis, and chondrogenesis was tested (Figure 1F). When mTDSCs were cultured in osteogenic medium for 28 days, Alizarin-red S positive calcium nodules were found in osteogenic induction group only but not in control group, in which mTDSCs were cultured in basic growth medium without osteogenic supplements. Lipid droplets were observed after adipogenic induction of mTDSCs for 14 days as indicated by Oil-red O staining whereas this was not observed in the control group. After culturing in chondrogenic induction medium for 21 days, the micromass formed by mTDSCs were stained positive for glycosaminoglycan (GAG)-rich matrix with Safranin O/Fast Green staining, which were negative in the control group. These data suggested that mTDSCs had multidifferentiation potential.

### Preparation and Characterization of the T-gel

**FIGURE 2:**
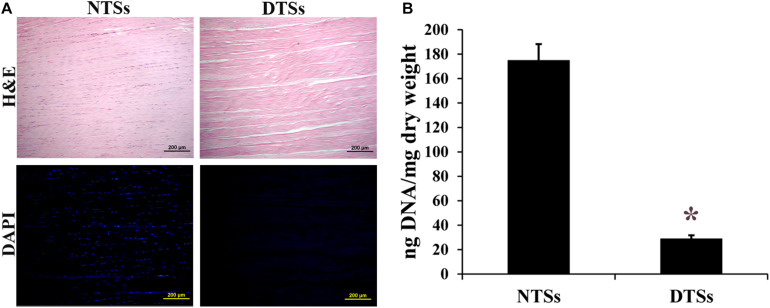
Assessment of decellularization effectiveness. **(A)** H&E staining and DAPI staining of the NTSs and DTSs. Scale bar = 200 μm. **(B)** PicoGreen analysis of residual DNA content in the NTSs and DTSs. *Signifies a *P-*value of <0.05 as compared to the NTSs.

Prior to preparation of the T-gel, the Achilles tendons from adult Macaca mulatta were subjected to complete decellularization. H&E staining of the tendon slices before and after decellularization demonstrated that preservation of the native tendon collagen structure while removing the cellular components (Figure 2A). DAPI staining also indicated an absence of nuclei in the DTSs. By comparison, abundant nuclear materials were observed in the NTSs (Figure 2A). DNA quantification assay indicated the residual DNA content was significantly reduced in the DTSs (28.62 ± 3.11 ng/mg) as compared with that in the NTSs (174.70 ± 13.42 ng/mg), which further demonstrated efficient removal of DNA from the native tendon (Figure 2B).

**FIGURE 3:**
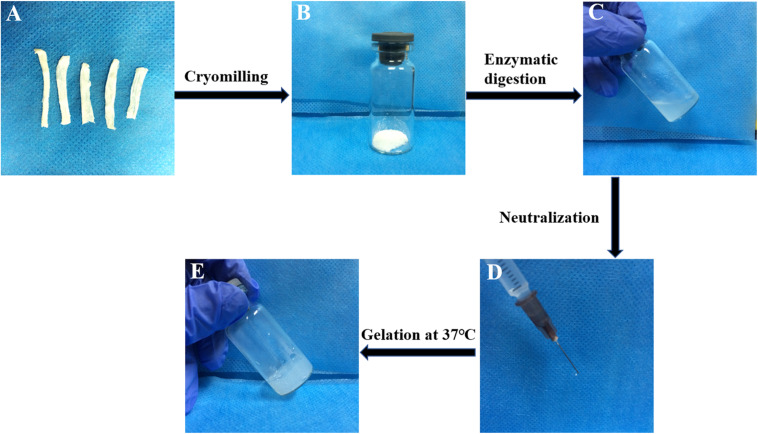
A typical process for the production of the T-gel from decellularized tendon ECM. The DTSs **(A)** were cryomilled to form powder **(B)**, subjected to enzymatic digestion **(C)**, and then neutralized **(D)** after dissolution, and incubated at 37°C to form the T-gel **(E)**.

A typical process for the production of T-gel is presented in Figure 3. The T-gel from decellularized tendon ECM was successfully prepared at 20 or 30 mg/ml concentration, which was determined to be injectable through a syringe with a 25 G needle. Specially, the higher concentrated T-gel (30 mg/ml) showed a stiffer structure and more stable gel-forming ability compared to the lower concentrated (20 mg/ml) T-gel. Therefore, 30 mg/ml T-gel was used in the following experiments.

**FIGURE 4:**
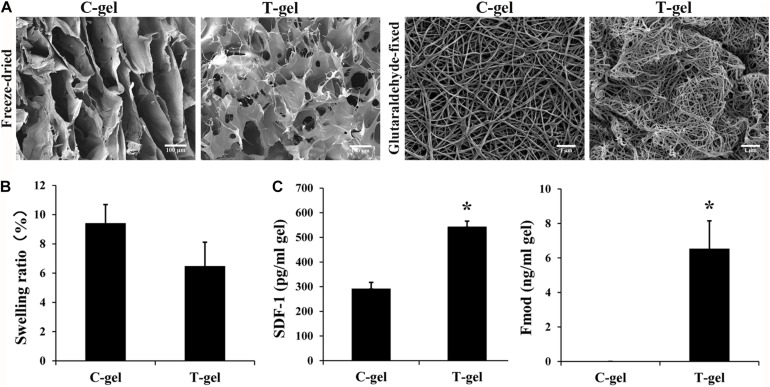
Analysis of microstructure, retained bioactive factors and equilibrium swelling properties of the C-gel and T-gel. **(A)** Representative SEM micrographs of the C-gel and T-gel. Scale bar = 100 μm for freeze-dried samples and Scale bar = 1 μm for glutaraldehyde-fixed samples. **(B)** Swelling ratios of the C-gel and T-gel. **(C)** The content of SDF-1 and Fmod in the C-gel and T-gel as measured by ELISA. *Signifies a *P*-value of <0.05 as compared to the C-gel.

For freeze-dried samples, SEM images of cross section of both gels exhibited an interconnected and highly porous structure. Notably, the C-gel showed interlaced lamellar and the T-gel showed alveolate (Figure 4A). For glutaraldehyde-fixed samples, SEM analysis revealed that both the C-gel and T-gel exhibited similar nanoscale collagen features (Figure 4A), with assembled nanofibers that were 87.77 ± 9.12 nm and 74.30 ± 5.33 nm in diameter, respectively.

The swelling ratio of the C-gel and T-gel is shown in Figure 4B. The T-gel showed a slightly lower swelling ratio than the C-gel, and no statistical significance was found between the two groups (*P* > 0.05).

The retention of the bioactive factors naturally existing in native tendon was demonstrated by the ELISA measures of protein extractions from the C-gel and T-gel. No detectable IGF-1 was found in the C-gel and T-gel (data not shown). SDF-1 and Fmod were present in the T-gel, while no detectable Fmod was present in the C-gel. The values of SDF-1 and Fmod in the T-gel were significantly higher than those in the C-gel (Figure 4C).

**FIGURE 5:**
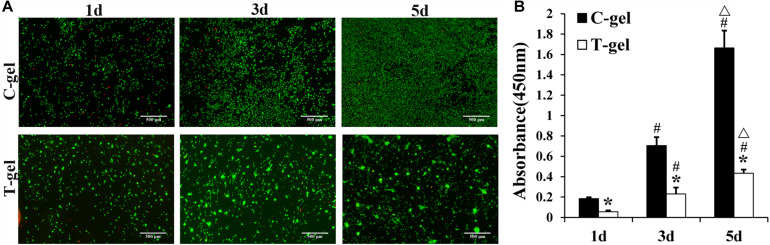
Cytotoxicity analysis of the C-gel and T-gel. **(A)** Live/Dead staining of NIH 3T3 fibroblasts seeded on the C-gel and T-gel at 1, 3, and 5 days by fluorescence microscopy (green, live cells; red, dead cells). Scale bar = 500 μm. **(B)** The cytotoxicity of the C-gel and T-gel using NIH-3T3 fibroblasts cultured for 1, 3, and 5 days as determined by the CCK-8 assay. *Signifies a *P-*value of<0.05 as compared to the C-gel; #, signifies a *P*-value of <0.05 as compared to 1 day for each gel; △, signifies a *P*-value of <0.05 as compared to 3 days for each gel.

The Live/Dead staining indicated that the majority of NIH-3T3 fibroblasts cultured on the surface of the C-gel and T-gel were viable, with few dead cells from 1 to 5 days (Figure 5A). However, fibroblasts cultured on the C-gel-coated plates showed more obvious increases in cell number than those on the T-gel-coated plates. Interestingly, fibroblasts were observed to infiltrate into the T-gel and tended to grow as cell clusters. The CCK-8 assay also verified significant lower cell viability of fibroblasts in the T-gel-coated group, as compared with that in the C-gel-coated group (*P* < 0.05, Figure 5B). Nonetheless, both groups showed cell number significantly increased with time in culture (*P* < 0.05, Figure 5B).

**FIGURE 6:**
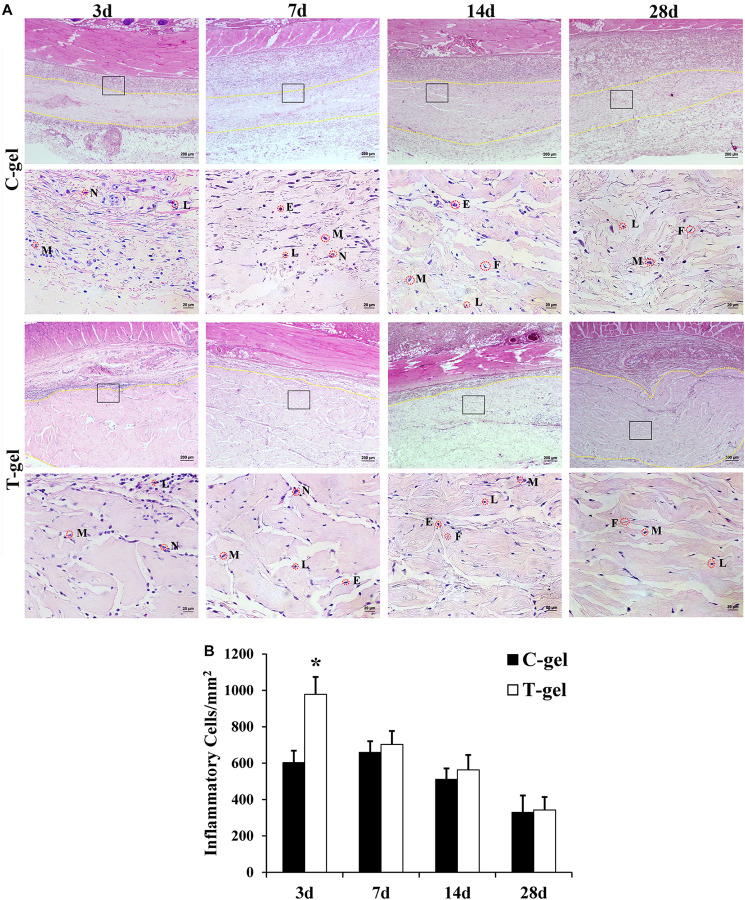
Histocompatibility evaluation of the C-gel and T-gel. **(A)** Histological appearance of tissue response to the C-gel and T-gel at 3, 7, 14, and 28 days post subcutaneous implantation in a rat model as revealed by H&E staining. Images in the bottom panels (Scale bar = 20 μm) are higher resolution images of the areas boxed in the images of top panels (Scale bar = 200 μm). M, macrophage; N, neutrophil; L, lymphocyte; E, eosinophil; F, fibroblast. **(B)** Semiquantitative evaluation of tissue response to the C-gel and T-gel at each time point. Total inflammatory cells were counted with H&E staining in five randomly selected 400× field-of-view and reported as average number per mm^2^. *Signifies a *P*-value of <0.05 as compared to the C-gel.

**FIGURE 7:**
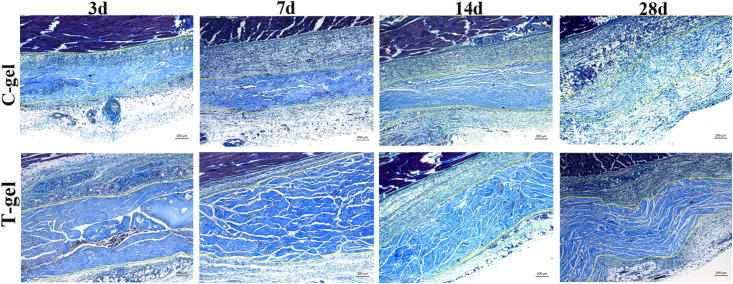
Masson’s Trichrome staining of the C-gel and T-gel at 3, 7, 14, and 28 days post subcutaneous implantation. Scale bar = 200 μm.

Histologically, at 3 days after injection, a large number of inflammatory cells appeared around the C-gel and T-gel, indicating that both gels elicited a moderate degree inflammatory response (Figure 6A). Quantitatively, the average inflammatory cells density in the T-gel group was significantly higher than that in the C-gel group at this time point (C-gel: 602.62 ± 65.68/mm^2^ vs. T-gel: 978.17 ± 95.36/mm^2^, *P* < 0.05, Figure 6B). At 7 days, numerous inflammatory cells, including macrophages, lymphocytes, neutrophils and eosinophils, infiltrated into the gel implants (Figure 6A). The number of inflammatory cells showed slight increase in the C-gel group but obvious decrease in the T-gel group. However, no significant differences in the average inflammatory cells density between the two groups were detected (C-gel: 659.39 ± 61.08/mm^2^ vs. T-gel: 703.06 ± 73.48/mm^2^, *P* > 0.05, Figure 6B). By 14 days, the infiltration of inflammatory cells gradually decreased and a small number of spindle-shaped fibroblasts were observed (Figure 6A). The average inflammatory cells density decreased to 510.92 ± 59.99/mm^2^ and 563.32 ± 81.91/mm^2^ for the C-gel and T-gel, respectively (*P* > 0.05, Figure 6B). By 28 days, the inflammatory reaction was basically eliminated along with the increase of fibroblasts (Figure 6A), and the number of inflammatory cells further decreased to 329.69 ± 92.44/mm^2^ and 342.79 ± 71.00/mm^2^ for the C-gel and T-gel, respectively (*P* > 0.05, Figure 6B). Besides, Masson’s Trichrome staining indicated no fibrous capsule formation at the interface between tissues and the gels (Figure 7). Both gels showed faint blue staining. The C-gel degraded more quickly than T-gel during the period of 28 days and tended to be much looser. Taken together, the T-gel did not elicit a significant immune response as compared to the C-gel with excellent biocompatibility.

### Functional Evaluation of the T-gel

**FIGURE 8:**
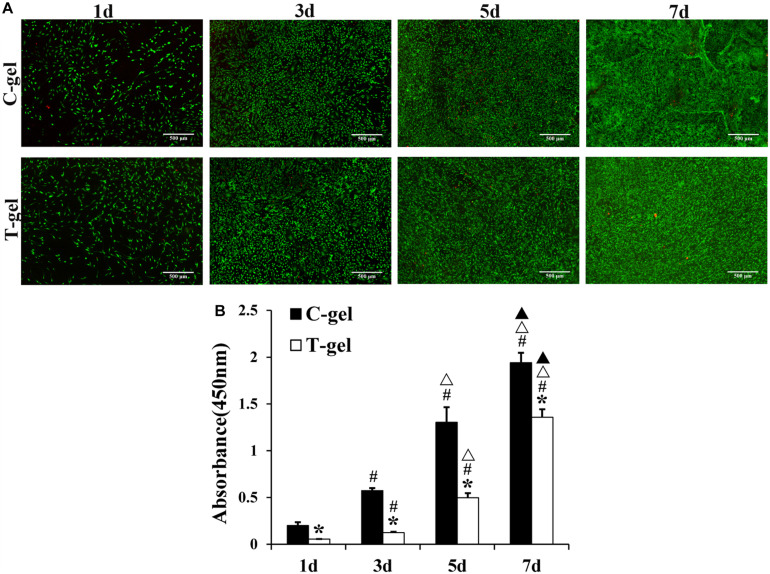
Cell proliferation assays of the C-gel and T-gel. **(A)** Live/Dead staining of mTDSCs seeded on the C-gel and T-gel at 1, 3, 5, and 7 days by fluorescence microscopy (green, live cells; red, dead cells). Scale bar = 500 μm. **(B)** Cell proliferation of mTDSCs cultured on the C-gel and T-gel for 1, 3, 5, and 7 days as determined by the CCK-8 assay. *, signifies a *P*-value of <0.05 as compared to the C-gel; #, signifies a *P-*value of <0.05 as compared to 1 day for each gel; △, signifies a *P*-value of <0.05 as compared to 3 days for each gel; ▲, signifies a *P*-value of <0.05 as compared to 5 days for each gel.

As shown in Figure 8A, mTDSCs were well attached on the surface of the C-gel or T-gel-coated plates after 1 day of culture. From 1 to 7 days, Live/Dead staining displayed negligible cell death on the C-gel and T-gel. Furthermore, both groups showed evident increase in cell number with time in culture. The CCK-8 assay indicated that significant lower cell viability of mTDSCs cultured on the surface of the T-gel at all time points, as compared with that of the C-gel (*P* < 0.05, Figure 8B). However, it’s worth noting that the number of mTDSCs from 1 to 7 days significantly increased with time (*P* < 0.05, Figure 8B). Overall, these results validated that the T-gel was able to support the survival and proliferation of mTDSCs.

**FIGURE 9:**
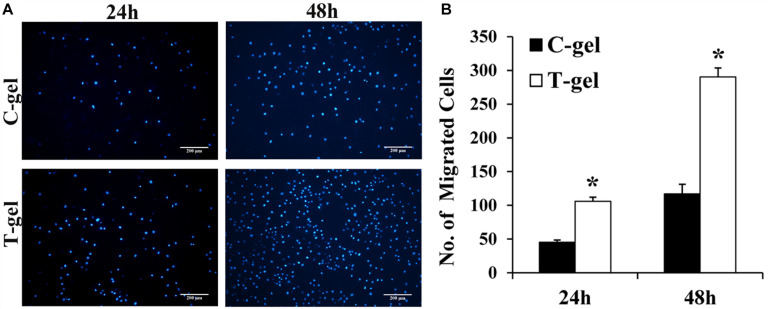
Cell migration assays of the C-gel and T-gel. **(A)** Representative fluorescence staining of the migrated mTDSCs for each group. Scale bar = 200 μm. **(B)** The semi-quantitative results of the number of the migrated mTDSCs for each group. *Signifies a *P*-value of <0.05 as compared to the C-gel.

DAPI staining of the migrated mTDSCs showed relatively more nuclei in the T-gel group than in the C-gel group at 24 or 48 h (Figure 9A). The semi-quantitative results of our Transwell migration assay revealed that the number of migrated mTDSCs in the T-gel group was significantly higher than that in the C-gel group at both time points (Figure 9B). In short, these results demonstrated the T-gel promoted the migration of mTDSCs.

**FIGURE 10:**
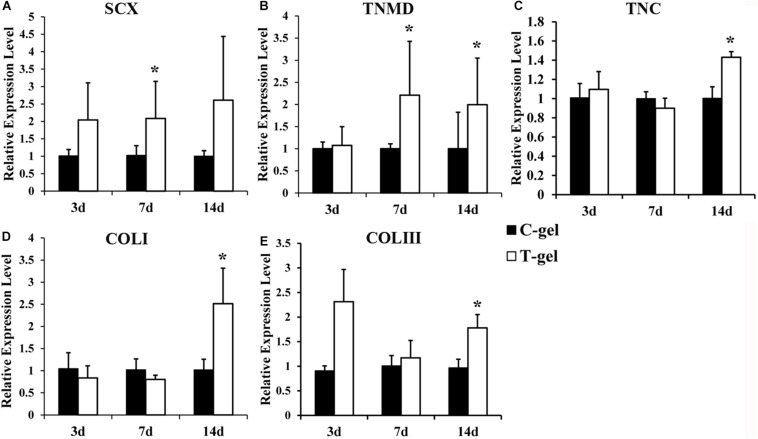
Cell differentiation assays of the C-gel and T-gel. **(A–E)** RT-qPCR analysis of tendon-related gene expressions of the mTDSCs seeded on the C-gel and T-gel at different time points. Gene expression levels are normalized to the housekeeping gene, GAPDH. *Signifies a *P-*value of <0.05 as compared to the C-gel.

Gene expression of mTDSCs cultured on the C-gel or T-gel-coated plates was quantified at the mRNA level by RT-qPCR. The expression of SCX was significantly up-regulated in mTDSCs cultured on the T-gel-coated group compared to those on the C-gel-coated group at 7 days but not at 3 or 14 days (Figure 10A). The expression of TNMD was significantly enhanced in mTDSCs cultured on the T-gel-coated group at 7 or 14 days when compared to those on the C-gel-coated group, and no significant difference was found at 3 days (Figure 10B). Though there was no significant difference between two groups at 3 or 7 days, the expression of TNC, COLI and COL III was elevated significantly at 14 days in mTDSCs cultured on the T-gel-coated group (Figures 10C–E). As a whole, these data indicated that the T-gel was capable of promoting tenogenic differentiation of mTDSCs.

## Discussion

The current study focused on demonstrating that the T-gel support the proliferation of mTDSCs and significantly promote the migration and tenogenic differentiation of mTDSCs compared to the C-gel. The isolated mTDSCs were first identified to have universal MSC characteristics. The DTSs from Macaca mulatta were verified to be thoroughly decellularized and the resultant T-gel exhibited highly porous structure or similar nanofibrous structure and approximately swelling ratio compared to the C-gel. More importantly, the T-gel was confirmed to retain some native tendon ECM bioactive factors, such as SDF-1 and Fmod. Furthermore, the T-gel was found to be cytocompatible with NIH-3T3 fibroblasts and displayed good histocompatibility when implanted into rat subcutaneous tissue. Collectively, these findings illustrate the effect of T-gel with nanofibrous structure and some bioactive factors of native tendon ECM microenvironment on the proliferation, migration and tenogenic differentiation of mTDSCs, as well as suggest the potential of the T-gel for treatment of tendinopathy or tendon repair and regeneration.

TDSCs, also described as tendon stem/progenitor cells (TSPCs), were isolated and identified for the first time in 2007 (Bi et al., 2007). After that, TDSCs, as a new member in MSC families, the roles of which in tendon metabolism, repair and regeneration have been studied extensively. Since 2007, TDSCs from human (Bi et al., 2007), mouse (Bi et al., 2007), rabbit (Zhang and Wang, 2010), rat (Rui et al., 2010), horse (Lovati et al., 2011), fetal bovine (Yang et al., 2016), and pig (Yang et al., 2018) were isolated and identified successively. In this study, we first isolated, identified, and used mTDSCs to explore the effect of T-gel on the proliferation, migration and tenogenic differentiation of stem cells. Our data demonstrated that the isolated tendon-derived cells from Macaca mulatta possessed universal MSC characteristics, including clonogenicity, the expression of stem cell markers and multidifferentiation potential. As far as we know, this is the first work that isolated and characterized Macaca mulatta TDSCs *in vitro*. The isolation and identification of mTDSCs are highly necessary as Macaca mulatta is highly similar to humans in terms of genetics and physiology and is the most widely used non-human primate in basic and applied biomedical research (Gibbs et al., 2007). Therefore, mTDSCs provide an attractive tool to study the physiological mechanism of tendinopathy and the basic tendon biology.

In the present study, the DTSs from Macaca mulatta Achilles tendons were fabricated using our previously established protocol (Ning et al., 2012), i.e., repetitive freeze/thaw, frozen section in combination with nuclease treatment 12 h, which was proved to be a mild and effective decellularization method and preserve almost all of the native tendon ECM microenvironment cues, after decellularization (Ning et al., 2015). The results of the current study showed that such decellularization method was effective for removal of the cellular and nuclear materials as evidenced by H&E staining and DAPI staining. DNA quantification assay also indicated such decellularization treatment significantly reduced the remaining DNA content. Previous studies showed repeated and intensive chemical decellularization of the porcine nerve significantly reduced DNA content but resulted in significant loss of ECM components, which led to the difficulty of hydrogel formation at 37°C (Lin et al., 2018). Lin et al. (2018) believed it was necessary to maintain the DNA content of porcine decellularized nerve matrix hydrogel between 40 and 50 ng/mg to ensure low immunogenicity and ease of gelatinization. However, our study showed it was easy to gelatinize when the DNA content of DTSs for preparation of the T-gel was only 28.62 ± 3.11 ng/mg. This discrepancy may be attributed to different tissue sources, different decellularization methods and different preparation process of ECM hydrogels.

ECM hydrogel formation is a collagen-based self-assembly process that is partly regulated by the presence of GAGs, proteoglycans, and other ECM proteins (Brightman et al., 2000; Saldin et al., 2017). In the present study, purified collagen type I from rat-tail was selected as the control due to the following reasons: (1) collagen type I is the most abundant and best studied collagen, and is also the main ECM component of tendons (Gelse, 2003); (2) collagen type I hydrogel possesses excellent biocompatibility, often used as three-dimensional substrates for cell culture *in vitro* and causes a very limited inflammatory reaction *in vivo* (Nöth et al., 2007; Antoine et al., 2014). After decellularization and solubilization treatments, the resultant T-gel samples that have been directly freeze-dried exhibited an interconnected and highly porous structure, which is closely similar to the C-gel, but not identical. More specifically, the C-gel showed interlaced lamellas while the T-gel showed alveolates. However, when the gel samples were fixed with glutaraldehyde and dehydrated by gradient alcohol, both the C-gel and T-gel exhibited similar nanoscale collagen features, with assembled nanofibers that were 87.77 ± 9.12 nm and 74.30 ± 5.33 nm in diameter, respectively. Our results are consistent with Brightman et al. (2000) who concluded that the fibrils of purified collagen type I exhibited a larger diameter compared to those of interstitial ECM. Liang et al. (2015) also reported that the average fiber diameter of collagen hydrogels was slightly higher than that of porcine small intestine submucosa ECM hydrogels (114 ± 4 nm vs. 105 ± 10 nm). Kreger et al. (2010) found the fiber structure data of collagen type I hydrogels at concentration above 3 mg/ml was notably absent. Antoine et al. (2014) stated that no research has been reported that present fiber structure analysis of collagen hydrogels when the collagen concentration exceeded 4 mg/ml, except for one article (Cross et al., 2010), which presented fiber structure images inconsistent with other publications. In this study, the concentration of C-gel was determined to be 2 mg/ml, which is also the maximum concentration recommended by the manufacturer. In fact, the nanofibrous structure has been demonstrated to promote stem cell differentiation (Zeng et al., 2014). In the present study, we speculated the significantly enhanced tenogenic differentiation of mTDSCs in the T-gel group is most likely caused by the bioactive factors retained in the T-gel, but we didn’t exclude the possible contribution of nanofibrous structure feature for the tenogenic differentiation of mTDSCs.

The T-gel developed in the present study retained some bioactive factors of native tendon ECM microenvironment, such as SDF-1 and Fmod. Unfortunately, no detectable IGF-1 was found in the C-gel and T-gel. However, our previous study demonstrated that more than 93% IGF-1 in native tendon was preserved in the canine DTSs using the same decellularization protocol (Ning et al., 2012). Hence, the loss of IGF-1 may be caused by the process of preparing the T-gel. As we all known, the loss of bioactive factors is almost inevitable in the process of preparing ECM hydrogels. Lin et al. (2018) found the loss of bioactive factors during the process of preparing the porcine decellularized nerve matrix hydrogel may lead to the suboptimal repair effect in rat sciatic nerve defect model. Encouragingly, our results from ELISA data indicated that the values of SDF-1 and Fmod in the T-gel were significantly higher than those found in the C-gel, which may account for higher ability of the T-gel to promote the migration and tenogenic differentiation of mTDSCs compared to that of C-gel. In the current study, we selected IGF-1, SDF-1 and Fmod for analysis because they may play important roles in promoting the proliferation, migration and tenogenic differentiation of stem cells. IGF-1, as one of insulin-like growth factors, has been demonstrated to regulate survival, proliferation, and differentiation of many types of cells, including stem cells (Bendall et al., 2007; Huat et al., 2014). SDF-1, as one of the most representative homing factors, has been extensively proved to stimulate stem cell homing (Shimode et al., 2009; Theiss et al., 2011; Chen et al., 2015) and migration (Jung et al., 2013; Park et al., 2017). Fmod, as one of critical components of TDSCs niches (Bi et al., 2007), was found to promote rat Achilles tendon repair *in vivo* and *in vitro* by gene delivery (Delalande et al., 2015). Our study also proved that soluble Fmod at an appropriate concentration can induce the tenogenic differentiation of TDSCs *in vitro* while inhibiting the chondrogenic and osteogenic differentiation of TDSCs (unpublished data). It should be pointed out that three representative bioactive factors were selected to detect in this study. There should be some other bioactive factors in the T-gel, which also may influence the migration and tenogenic differentiation of mTDSCs.

To apply the T-gel as a carrier of cells or growth factors or directly used for tendon repair and regeneration, the cytotoxicity and histocompatibility of T-gel were evaluated. As expected, the C-gel as a commercial product specifically used for 2D and 3D cell cultures showed excellent cytocompatibility. The results of Live/Dead staining revealed that the majority of NIH-3T3 fibroblasts were viable in a time-frame of 5 days when cultured on the surface of both gels. Yet, fibroblasts cultured on the surface of the C-gel showed more obvious increases in cell number than those on surface of the T-gel. The CCK-8 test also displayed a similar trend. It must be admitted that the cytocompatibility of the T-gel was indeed inferior to that of the C-gel, suggesting that the method of preparing the T-gel need to be further optimized. After all, the DTSs were demonstrated to facilitate the proliferation of NIH-3T3 fibroblasts in our previous study (Ning et al., 2012). Nonetheless, compared with the C-gel, the T-gel was deemed to be cytocompatible with NIH-3T3 fibroblasts because the number of viable cells within the T-gel from 1 to 5 days was significantly increased with time. Interestingly, NIH-3T3 fibroblasts were observed to infiltrate into the T-gel and tended to grow as cell clusters. Labuschagne et al. (2019) found that cell clustering-induced metabolic switch promotes metastatic capacity. Hence, we speculated NIH-3T3 fibroblasts infiltrated into the T-gel with the formation of cell clusters, which led to decreased cellular proliferation and increased antioxidant defense. Hopefully, when implanted into rat subcutaneous tissue, the T-gel, as good as the C-gel, displayed good histocompatibility. Both gels invoked just a moderate degree of inflammation at early time points. Along with the degradation of both gels, the inflammation reaction gradually subsided over time. In addition, the two types of gels, especially the C-gel, significantly degraded over time. This is consistent with the findings from previous study which reported the collagen carrier is absorbed within 1 month after injection (Lemperle et al., 2010). Collectively, these results demonstrated that the T-gel was biocompatible.

The mechanisms of the ECM hydrogels mediating cell behavior are not fully understood. Saldin et al. (2017) came up with the native ECM microenvironment signals that are preserved during production of a tissue-specific ECM hydrogel may markedly affect cell viability, proliferation, migration, morphology, differentiation and phenotype. Unlike hydrogels formed by single ECM component, ECM hydrogels preserve most of the biochemical complexity of native ECM, which may regulate stem cells fate in a tissue-specific manner. To our knowledge, no studies have clarified the effect of decellularized tendon hydrogel upon stem cell behavior, involving the proliferation, migration and tenogenic differentiation. In the current study, although not as good as the C-gel, the T-gel was found to support the proliferation of mTDSCs, as evidenced by the high cell viability and very obvious proliferation of mTDSCs on the surface of the T-gel in a time-frame of 7 days. What needs to be pointed out is that we used the same detection methods including the Live/Dead staining and CCK-8 assay to evaluate the cytotoxicity of T-gel and the effect of T-gel on stem cell proliferation, respectively. An interesting finding is that mTDSCs seeded on the surface of the T-gel showed higher vitality and faster proliferation than NIH-3T3 fibroblasts. Further research is needed to determine the underlying causes. Another encouragingly, consistent with the results from ELISA data, the results of our Transwell analysis and RT-qPCR analysis revealed that the T-gel significantly promote the migration and tenogenic differentiation of mTDSCs compared to the C-gel, respectively. More specifically, in comparison with the C-gel, the T-gel with significant higher value of SDF-1 markedly promote the migration of mTDSCs at both time points, which is in accordance with the findings of Chen et al. (2015) who found the addition of SDF-1 to the radially oriented collagen scaffold further accelerated the migration of bone marrow-derived MSCs. As expected, the T-gel as a tendon-specific ECM hydrogel, which retained nanofibrous structure and some bioactive factors (SDF-1, Fmod et al) of native tendon ECM microenvironment, significantly promoted tenogenic differentiation of mTDSCs based on the tendon-related gene expressions. SCX, as a well-known tendon marker, was significantly up-regulated in the T-gel group compared to that in the C-gel group at 7 days. Although a previous study suggested that the expression of SCX was not specific for adult tendon (Jelinsky et al., 2010), SCX was still often selected as one of tendon-related markers in many studies (Yang et al., 2013; Czaplewski et al., 2014; Yin et al., 2016). It has been reported that SCX positively regulates the expression of TNMD (Shukunami et al., 2006). Consistently, the significantly up-regulated expression of TNMD in the T-gel group was found at 7 and 14 days. TNC, as an ECM glycoprotein, is known to be existing in native tendon and involved in the regulation of collagen fibrillogenesis (Burk et al., 2014). Though TNC was found to be expressed in diverse cell types and its upregulation is also explicitly related to non-musculoskeletal diseases (Brellier et al., 2009), it was also frequently used as an additional tenogenic differentiation marker (Kuo and Tuan, 2008; Yang et al., 2013). In this study, we found that the T-gel significantly up-regulated the expression of TNC at 14 days compared to the C-gel. Additionally, the expressions of other two tendon-related markers COL I and COL III were also significantly higher in the T-gel group at 14 days. In summary, the T-gel was verified to promote the tenogenic differentiation of mTDSCs.

There are several limitations in this study. First, the method of preparing the T-gel needs to be further optimized to obtain excellent cytocompatibility as good as the commercialized C-gel and ensure a long shelf life for potential clinical use. Second, although there may be many different types of bioactive factors preserved in the T-gel, only three representative factors including IGF-1, SDF-1 and Fmod were investigated. In future study, we should pay attention to more kinds of bioactive factors. Third, an *in vivo* study is needed to verify the effectiveness of the T-gel for promoting tendon healing.

In conclusion, our study demonstrates that TDSCs derived from Macaca mulatta have universal MSC characteristics and the T-gel derived from Macaca mulatta decellularized tendon is capable of supporting the proliferation of mTDSCs as well as promoting the migration and tenogenic differentiation of mTDSCs. Our findings indicated that the T-gel, with its retained nanofibrous structure and some bioactive factors of native tendon ECM microenvironment, represents a promising hydrogel for tendon regeneration.

## Data Availability Statement

The original contributions presented in the study are included in the article/supplementary material, further inquiries can be directed to the corresponding author/s.

## Ethics Statement

The animal study was reviewed and approved by the Institutional Animal Care and Use Committee (IACUC) in Sichuan University.

## Author Contributions

L-JN and Ya-JZ conceived the study and performed the majority part of the experiments and data interpretation, drafted and edited the manuscript. Yan-JZ and MZ provided help for the experiments. WD provided resources and J-CL provided help for the investigation. T-WQ conceived and designed the study, and critically reviewed the manuscript. All authors have read and approved the final submitted manuscript.

## Conflict of Interest

The authors declare that the research was conducted in the absence of any commercial or financial relationships that could be construed as a potential conflict of interest.
